# Design and baseline data in the BAriatic surgery SUbstitution and Nutrition study (BASUN): a 10-year prospective cohort study

**DOI:** 10.1186/s12902-020-0503-z

**Published:** 2020-02-14

**Authors:** Gudrún Höskuldsdóttir, Karin Mossberg, Ville Wallenius, Angelos Al Nimer, Wiveka Björkvall, Sören Lundberg, Carl-Johan Behre, Malin Werling, Björn Eliasson, Lars Fändriks

**Affiliations:** 1 Institute of Medicine, University of Gothenburg, Gothenburg, Sweden; 2 Department of Medicine, Sahlgrenska University Hospital, 413 45 Gothenburg, Sweden; 3 Institute of Clinical Sciences, University of Gothenburg, Gothenburg, Sweden; 4 Department of Surgery, Sahlgrenska University Hospital, Gothenburg, Sweden; 5 Department of Cardiology, Sahlgrenska University Hospital, Gothenburg, Sweden; 6 Department of Public Health and Community Medicine, Sahlgrenska University Hospital, Gothenburg, Sweden

**Keywords:** Obesity, Bariatric surgery, Diet, Prospective study, Cohort study

## Abstract

**Background:**

There is still a lack of knowledge on long-term effects of surgical and non-surgical weight-lowering treatments. BASUN is a prospective study with 10 years of follow-up that will observe the effects and consequences of surgical and medical treatment of obesity. The aims are to cover areas where data on long-term outcomes are lacking, e.g., nutritional deficiencies, substance abuse, psychiatric health, as well as patient-reported outcomes.

**Methods:**

BASUN is a cohort study that recruited study persons with obesity (BMI ≥ 35 kg/m^2^) referred to the Regional Obesity Centre of Region Västra Götaland. The interventions were Roux-en-Y gastric bypass (RYGB) or Sleeve gastrectomy (SG), or 12 months of structured, multi-professional medical treatment (MT), including very low energy diet, followed by diet and pharmaceutical treatment. The study is not randomized, but based on patients preferences and multidisciplinary assessments. The study persons are examined at baseline, 2, 5, and 10 years with blood tests, measurements and questionnaires. The recruitment period lasted from May 2015 to November 2017.

**Results:**

One thousand one hundred twenty-seven patients were included (74% female). Three hundred eighty-two patients were accepted for medical treatment, 589 for surgical treatment (388 RYGB and 201 SG) and 156 patients left the study without treatment, leaving a final study population of 971 patients. There were slight differences between the treatment groups with regards to age and BMI. Pharmaceutical treatments, level of education, smoking and marital status were not significantly different between the groups.

**Conclusion:**

This study will follow 971 obese subjects in clinical practice treated with the best surgical or medical methods currently available. It has the potential to evaluate outcomes usually not reported in short-term studies, and to assist in identifying factors that are of importance for the choices of treatment. The main limitations are non-randomization and differences in baseline characteristics. The large number of participants and the length of the prospective follow-up are major strengths of the study. BASUN is designed to identify both early and late benefits and adverse events of treatment of obesity*.*

**Trial registration:**

This trial was prospectively registered on March 03, 2015; NCT03152617.

## Background

Bariatric surgery is an effective treatment of obesity as demonstrated by prospective cohort control studies, randomized control trials, and observational register-based studies [1–4]. In addition to sustained weight loss and improved well-being, comorbidities like type 2 diabetes improve after bariatric surgery, and risk of cardiovascular disease and premature death have been shown to be reduced when compared to conventional interventions, although there is also a panorama of potential side effects [5].

Despite the amount of literature focusing on effects of obesity treatments, the gap of knowledge on long-term complications remains. For example nutritional deficiencies, substance abuse, psychiatric as well as patient-reported outcomes, might lead to unexpected and increased costs for society, along with suffering for the affected individual. In addition, it is unclear which surgical method has better long-term effects on weight and comorbidities. Furthermore, how do these surgical techniques compare to medical treatment in obese persons, who frequently exhibit co-morbidities and psychosocial conditions [6].

BASUN is a prospective study with 971 participants and 10 years of follow-up, that was initiated to observe the consequences of bariatric surgery and to compare these to effects of medical (non-surgical) treatment of obesity. The setting is the Regional Obesity Centre of Region Västra Götaland, through which obesity treatment in an eighth of the Swedish population is coordinated. Patients with obesity are a heterogenic group and choosing the most suitable treatment option can be challenging. The main aim of the study is to further assist in choosing the most beneficial treatment option for each patient without causing harm. The study covers a range of areas where significant data on long-term outcomes are lacking. The primary focus will be on comparing nutritional status after surgical and medical treatment. Secondary outcomes include changes in body composition, progression of psychiatric disorders, gastrointestinal symptoms, eating disorders, quality of life, levels of physical activity, effects of socioeconomic status and health economics. Some subjects will also participate in sub-studies addressing effects on bone metabolism or oral health.

The BASUN study will evaluate outcomes usually not reported in minor short-term studies, and describe groups of individuals at risk of long-term complications after treatment of obesity. The overall goal is to contribute to an improvement of the care and to reduce long-term adverse outcomes of surgical and medical treatment of obesity, and to define groups of individuals who demand further health care efforts before and after the interventions. This may result in a more equal health care for patients and the best possible use of public health care resources. In this paper we describe the design of the study as well as the baseline characteristics of the study population.

## Methods

The BASUN study is a prospective non-randomized cohort study conducted in clinical practice, expanded with sampling of stool specimens and blood, questionnaires and data available in official registries and databases. We recruited study subjects by referral from the primary health care centres. All referrals for obesity treatment in Region Västra Götaland, Sweden, are centrally coordinated through a regional referral body, the Regional Obesity Centre (ROC), located at Sahlgrenska University Hospital in Gothenburg. The recruitment started in May 2015 and closed in November 2017. All persons willing and able to receive verbal and written information in Swedish about the study were invited after attending information meetings. The study subjects are planned for follow-up examinations at 2, 5, and 10 years.

### Obesity treatment

Criteria for bariatric surgery in public healthcare in Sweden are in essence similar to those advocated in international guidelines, including BMI > 35 kg/ m^2^ with obesity-related comorbidities such as diabetes and sleep apnoea, or BMI > 40 kg/m^2^ without comorbidities [7]. Contraindications include drug or alcohol abuse, unstable psychiatric disorders, age under 18 years, cancer during the last 5 years, or poor general health condition [8]. A multidisciplinary board assesses the eligibility of all persons to undergo surgical treatment. Only Roux-en-Y gastric bypass (RYGB) or sleeve gastrectomy (SG) are used as the primary surgical procedure in the public health care in Sweden. Laparoscopic antecolic antegastric RYGB is carried out as previously described [9, 10]. Laparoscopic SG is carried out in line with state-of-the art principles described at the Fifth International Consensus Conference on SG [11]. The final choice of surgical method to be used in each individual case was made in consensus between the operating surgeon and the patient, taking into consideration the patients’ preference as well medical conditions. Postoperative follow-up is conducted in accordance with Nordic guidelines for follow-up and dietary supplementation after bariatric surgery [12]. The operating unit conducts follow-up at 6 weeks, 6 and 12 months after surgery, then the patients are referred to their primary health care units that continue to do yearly follow-ups. All patients are prescribed dietary supplementation with iron 100 mg/day, vitamin B12 1 mg/day, calcium 500 mg and vitamin D 800 U-combinations twice daily, and multivitamin and mineral preparations containing at least 1,4 mg thiamine, 400 μg folate and 14 mg zinc, twice daily. During the first 2 months after surgery all operated patients were prescribed proton pump inhibitors, e. g. omeprazole 20 mg once daily.

Medical treatment is offered to all persons 18 years of age or older with BMI > 35 kg/ m^2^, who do not qualify, or are not willing to undergo surgical treatment. If there are no contraindications, such as binge eating, severe psychological disturbance or medical condition, the intervention starts with a very low energy diet (VLED) period for 12, 16 or 20 weeks depending on the starting weight (BMI 35–39.9, 40–49.9 or ≥ 50 kg/m^2^, respectively). During this period the daily intake is 450–800 kcal as well as a recommended 1.5–2 L of fluids per day. The patients have appointments with a nurse at the start and then after 2, 5, 8 and 12 weeks.

The VLED period is followed by a period of food reintroduction lasting 12 weeks guided and monitored by a dietitian or clinical nutritionist. Each meal has an energy content of 300–475 kcal and for every meal that was introduced monthly (breakfast, lunch, dinner) a VLED meal was removed. To estimate the energy need for each individual for weight reduction, the Harris Benedict sex-specific equations are used multiplied with a physical activity level of 1.3 to 1.4 and minus 30% representing the estimated energy deficit to achieve weight reduction [13]. After the food reintroduction period the patients continues with such energy-restricted diet. The dietary advice is based on the Nordic Nutrition Recommendation and each patient receives written instructions for a diet of 1400–1600 kcal per day (15–20% energy percent (E%) protein, 30 E% fat and 50–55 E% carbohydrates) that was divided into three to five meals [13]. During the food reintroduction period, the patients had monthly appointments with a dietician for the remainder of the 12-month treatment period. The patients have appointments with a physician after 6 months and 12 months for addition of GLP-1 receptor agonists, SGLT-2 inhibitors, orlistat, or a combination of bupropion and naltrexone when appropriate as well as assessment of blood tests. Advice on physical activity was given by all professions at all visits.

### Demographic data and blood samples

Demographic data at baseline was collected. This includes age, gender, civil status, working condition, education, community, country of birth, medication, comorbidities, smoking habits, and a 4-days dietary recall. Physical measurements were conducted regarding height and weight. Certain blood samples were analysed immediately, while additional blood samples were collected and stored in a biobank until future analysis. Results from blood work that was collected before the start of treatment was gathered from electronic medical records. Information on blood tests and measurements are given in Table 1.

**Table 1:** Blood tests and measurements

Blood samples for direct analysis	Collected from medical records	Blood samples frozen for later analysis
Complete blood cell counts (White blood cell count, red blood cell indices, hemoglobin, erythrocyte and reticulocyte counts, hematocrit)^a^	Liver function (ALAT, ASAT, bilirubin)^a^	1 EDTA 4 ml^a^ 1 EDTA, 8,5 ml^a^ 2 serum, 8,5 ml^a^ 1 Lithium heparin, 8,0 ml^a^
Erythrocyte Sedimentation Rate^a^	Kidney function (serum creatinin, estimated glomerular filtration rate, GFR)^a^	
Plasma glucose and HbA1c^a^	Blood lipids (HDL and LDL cholesterol, triglycerides)^a^	
Ionized calcium^a^	Thyroid function (fT4, TSH)^a^	
Stool samples^b^		

^a^At baseline, 2 years, 5 years and 10 years

^b^At baseline and 6 months

### Self-evaluated validated questionnaires

Specific questionnaires are completed by the study participants at baseline and will be completed at 2, 5 and 10 years of follow-up as shown in Table 2. The questionnaires cover three areas: gastrointestinal symptoms and eating problems, physical activity and quality of life, and psychological health.

**Table 2:** Questionnaires

Self-evaluated questionnaire	Outcome	Reference
Bristol stool form scale (BSFS)	Bowel habits	Patel et al. 2015 [14]
Rome III criteria	Functional gastrointestinal disorders	
21-item Three Factor Eating Questionnaire (TFEQ-R21)	Eating behavior	Cappelleri et al. 2009 [15]
Questionnaire on Eating and Weight Patterns-Revised (QEWP-R)	Binge eating disorder	Borges et al. 2005 [16], Dorflinger et al. 2017 [17]
“Saltin Grimby” physical activity level scale	Physical activity	Grimby et al. 2015 [18]
RAND-36 item healty survey	Health-related quality of life	Krops et al. 2018 [19]
EuroQol five-dimensional questionnaire (EQ-5D)	Health-related quality of life	Sullivan et al. 2016 [20]
Becks Anxiety Inventory (BAI)	Symptoms of anxiety	Steer et al. 1997 [21]
Patient Health Questionnaire-9 (PHQ-9)	Symptoms of depression	Kroenke et al. 2001 [22]
ADHD Self Reporting Scale (ASRS)	ADHD screening	Kessler et al. 2005 [23]
Wender Utah Rating Scale (WURS)	Distinguish ADHD from other psychological disorders	Ward et al. 1993 [24]
Alcohol Use Disorders Identification Test (AUDIT)	Alcohol consumption	Babor et al. 2001 [25]
Drug Use Disorders Identification Test (DUDIT)	Drug related-problems	Berman et al. 2003 [26], Hildebrand et al. 2015 [27]

*ADHD* Attention deficit hyperactivity disorder

#### Assessment of gastrointestinal health and eating disorders

We use four different questionnaires to investigate gastrointestinal symptoms and eating problems. The Bristol stool form scale (BSFS) evaluates bowel habits of all participants [14]. This is considered a useful marker for colonic transit. The Rome III criteria will be used to detect functional gastrointestinal disorders in our study. The 21-item Three Factor Eating Questionnaire (TFEQ-R21) is a scale with which three domains of eating behaviour are measured: cognitive restraint, uncontrolled eating and emotional eating [15]. It is used both in obese and non-obese individuals. The Questionnaire on Eating and Weight Patterns-Revised (QEWP-R) is a 27-item self-administered questionnaire for the diagnosis of binge eating disorder [16, 17].

#### Assessment of levels of physical activity and quality of life

Three different questionnaires are used to investigate physical activity and quality of life. The “Saltin Grimby” physical activity level scale is a four-level questionnaire used to assess physical activity in leisure time [18]. The RAND-36 is a widely used survey which assesses health-related quality of life [19]. EuroQol five-dimensional questionnaire (EQ-5D) is a standardized instrument designed to measure health related quality of life in various health conditions and treatments [20]. EQ-5D comprises five dimensions: mobility, self-care, everyday activities, pain/discomfort and anxiety/depression.

#### Assessment of psychological health and substance abuse

We also use four different questionnaires to investigate the psychological health among the study persons. Becks Anxiety Inventory (BAI) will measure the severity of anxiety in the study participants [21]. The Patient Health Questionnaire-9 (PHQ-9) is a self-reported measure of depression consisting of nine items matching the DSM-IV criteria of major depression, which will be used to detect depressive symptoms among the study participants [22]. To screen for ADHD, the ADHD Self Reporting Scale (ASRS) is used. ASRS was developed in collaboration with the World Health Organization (WHO) [23]. In addition, the Wender Utah Rating Scale (WURS) is used, that retrospectively assess childhood ADHD symptoms, to distinguish ADHD from disorders that present symptoms that overlap with ADHD symptoms such as bipolar disorder and borderline personality disorder [24]. Alcohol Use Disorders Identification Test (AUDIT), is used to identify individuals with harmful patterns of alcohol consumption. This was developed by WHO in 1992 [25]. Drug Use Disorders Identification Test (DUDIT) is used to screen individuals for drug related-problems. This was developed in Sweden in parallel to AUDIT and has been further evaluated [26, 27].

Within the study, there is a possibility to collect patient data from Swedish databases to be linked to data in the BASUN study. These databases are shown in Table 3 [1, 28–32].

**Table 3:** Swedish databases linked to study

Databases	Specific area	Reference
Scandinavian Obesity Surgery Register	Obesity post bariatric surgery	Hedenbro JL et al. 2015 [28]
The National Diabetes Register	Diabetes Mellitus	Eliasson B et al. 2015 [1]
Swedish National Inpatient Register	Diagnosis of diseases	Ludvigsson JG et al. 2011 [29]
Swedish Child Health Care Register	Child healthcare	Emilsson et al. 2015 [30]
Swedish Prescribed Drug Register	Medication	Wettermark et al. 2007 [31]
Swedish Fracture Register	Nonspinal skeleton injury	Wennergren et al. 2015 [32]
Swedish Social Insurance Agency	Sick leave	www.government.se/government-agencies/social-insurance-agency%2D%2Dforsakringskassan
Swedish Public Employment Service	Employment	www.government.se/government-agencies/swedish-public-employment-service/
Statistics Sweden (SCB)	Public statistics	www.scb.se/en

### Statistical analysis

In this report descriptive statistics were calculated using IBM SPSS 25.0. Continuous variables are reported as median with ranges as the variables were not normally distributed according to Shapiro Wilk test and visual inspection of boxplots. Categorical variables are reported as numbers (n) and proportions (%). Kruskal-Wallis test was used for comparison of age, BMI, HbA1c, HDL and LDL cholesterol between the four treatment groups and Fisher’s exact test for comparison of gender, marital status, education, smoking and pharmaceutical treatment. Further analysis of age and BMI were done using Mann Whitney U testing. The level of significance was set at *p* < 0.05.

In future analyses of the data, Cox regressions and machine learning techniques will we used to evaluate importance of baseline conditions and characteristics for the long-term results in the three study groups.

## Results

**Fig. 1:**
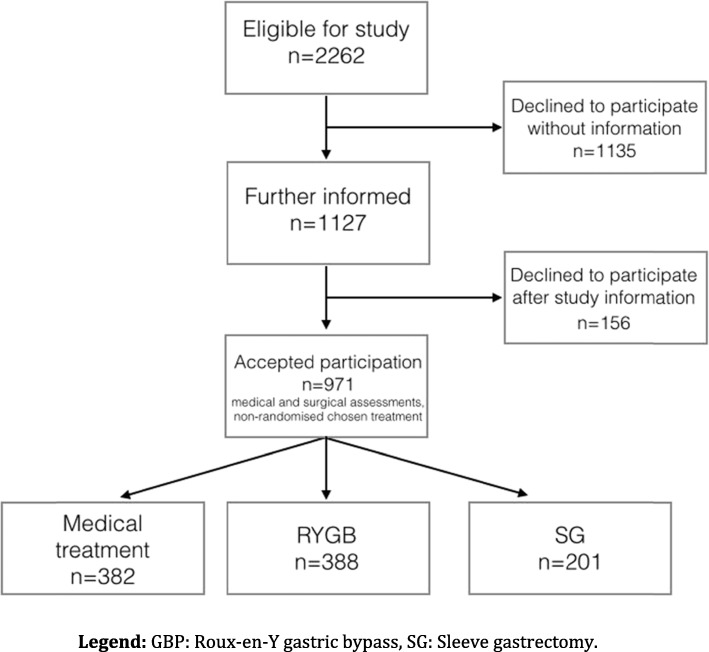
Participants. GBP: Roux-en-Y gastric bypass, SG: Sleeve gastrectomy

**Fig. 2:**
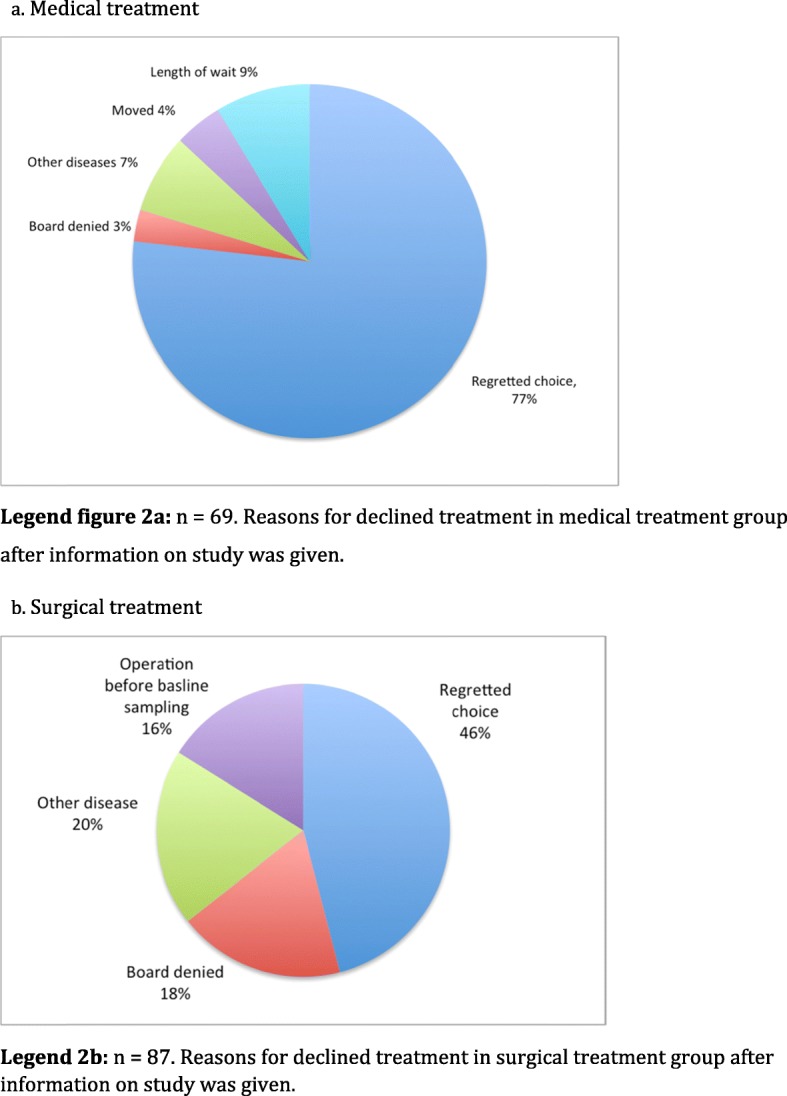
Patients that declined to participate in study. 2a. Medical treatment. **a** *n* = 69. Reasons for declined treatment in medical treatment group after information on study was given. **b** Surgical treatment. *n* = 87. Reasons for declined treatment in surgical treatment group after information on study was given

One thousand one hundred twenty-seven patients were invited to participate in the study, 832 females (73,8%). Three hundred eighty-two patients were accepted for medical treatment, 589 for surgical treatment (388 RYGB and 201 SG) and 156 patients left the study without treatment. That left a study population of 971 patients, as described in Fig. 1. Clinical characteristics and treatments of the individuals in the three groups at baseline are given in Table 4. There were possible differences between the groups with regards to age and BMI. The differences were apparent between medical treatment group and gastric bypass group as well as between the medical group and the gastric sleeve group according to Mann Whitney U analysis. There were no significant differences between the groups regarding distribution of sex, education, pharmaceutical treatment for diabetes, hypertension, hyperlipidemia, pain, anxiety and depression, gastric reflux, hypothyroidism, attention deficit hyperactivity disorder or treatment for deficiency of iron, vitamin B12, vitamin D, folic acid and calcium. The differences between the groups with regards to treatment with antipsychotics, smoking, and marital status were not significant either. When examined, there were differences in levels of high-density lipoproteins but the groups were similar with regards to levels of low-density lipoproteins. Further analysis of the group that left the study without starting treatment can be seen in Fig. 2a and b.

**Table 4:** Baseline characteristics

	MT (*n* = 382)	RYGB (*n* = 388)	SG (*n* = 201)	*P*
Female	276 (72,3)	301 (77,6)	152 (75,6)	0.206
Age (years)	49.0 (18–78)	43.5 (18–62)	41 (18–63)	< 0.0001
BMI (kg/m^2^)	40.1 (31.6–90.4)	41.8 (34.8–65.3)	41.6 (35–63.7)	< 0.0001
HbA1c (mmol/mol)	36 (25.0–95.0)	37 (26–118)	36 (27–73)	0.173
HDL (mmol/l)	1.3 (0.6–2.6)	1.2 (0.4–2.3)	1.2 (0.7–2.0)	0,003
LDL (mmol/l)	3.1 (1.2–6.7)	3.2 (0.8–6.2)	3.3 (0.9–5.7)	0.627
Glucose lowering treatment	55 (14,4)	55 (14,2)	28 (13,9)	0.990
Blood pressure lowering treatment	135 (35,3)	112 (28,9)	58 (28,9)	0.109
Lipid lowering treatment	52 (13,6)	51 (13,1)	23 (11,4)	0.768
Treatment for anxiety/depression	85 (22,3)	68 (17,5)	52 (25,9)	0.101
Treatment with antipsychotics	13 (3,4)	4 (1,0)	6 (3,0)	0.078
Pain medication	80 (20,9)	63 (16,2)	38 (18,9)	0.238
Hypothyroidism treatment	49 (12,8)	42 (10,8)	26 (12,9)	0.613
ADHD treatment	8 (2,1)	2 (0,5)	2 (1)	0.124
Iron supplements	5 (1,3)	3 (0,8)	3 (1,5)	0.685
B-vitamin supplements	14 (3,7)	9 (2,3)	9 (4,5)	0.313
D-vitamin supplements	3 (0,8)	7 (1,8)	4 (2,0)	0.347
Folic acid supplements	6 (1,6)	4 (1,0)	6 (3,0)	0.221
Calcium supplements	6 (1,6)	1 (0,3)	0 (0)	0.055
Protonpump inhibitor treatment	50 (13,1)	39 (10,1)	30 (14,9)	0.180
Education				0.082
Elementary	51 (13,4)	42 (10,8)	15 (7,5)	
Secondary	141 (36,9)	199 (51,3)	98 (48,8)	
Tertiary	127 (33,2)	101 (26,0)	69 (34,3)	
Smoking	23 (6,0)	24 (6,2)	13 (6,5)	0.884
Married/cohabitation	206 (53,9)	242 (62,4)	121 (60,2)	0.955

Data are n (%) or median (range)

*MT* Medical treatment, *GBP* Roux-en-Y gastric bypass, *SG* Sleeve gastrectomy, *BMI* Body mass index, *HDL* High-density lipoprotein, *LDL* Low-density lipoprotein

## Discussion

The BASUN study will follow 971 obese subjects treated with the best surgical and medical methods currently available for up to 10 years in clinical practice. We here present a description of the study methodology as well as baseline characteristics of the study population.

The size of the study population and the length of follow-up are clear strengths of the study. This large cohort is representative for this group of patients in the Western region of Sweden with a population of approximately 2 million individuals since the treatment of severe obesity is centralized in this part of the country. Another major advantage of the study is the access to national databases to collect patient information which simplifies follow-up and reduces risk for unexplained drop outs. BASUN includes all patients that meet criteria for the treatment of severe obesity, not only focusing on patients with established comorbidities as diabetes, as in many other studies, e.g., the STAMPEDE study [33]. BASUN also does not only compare the effects of medical treatment and surgery, but also the two most commonly used surgical methods used today, Roux-en-Y gastric bypass and sleeve gastrectomy.

The fact that the patients were not randomized to different treatment options can be seen as a limitation of the study. Randomizing the patients was not possible as we wanted to compare outcomes of the treatments for all obese subjects at our centre in clinical practice, also for those that were not primarily interested in or hade contraindications for surgical treatment. Patient involvement in the choice of treatment is representative for real life and can also be seen as a strength of the study. This also explains why the groups differ slightly at baseline. In our practice, we are more restrictive with offering surgery to patients above 60 years of age due to the suggested higher risk of complications and presumably lower benefit. Older patients can be accepted for medical treatment and this explains the slight difference in age between the medical group and the two surgical groups. The groups were similar with regards to pharmaceutical treatment, including treatment with vitamins and minerals, which indicates a comparable status of patient health before the start of treatment. Levels of education, marital status and smoking were similar between the groups and should therefore not be confounding factors. Within the scope of BASUN, we do not follow persons with language barriers, or unwillingness to participate, which could affect the interpretation of future results.

The clinical characteristics of the BASUN cohort resembles the participants of the Swedish Obese Subjects (SOS), which is a study that has been crucial to the widespread use of obesity surgery [34]. Many comparative studies have focused on intensive lifestyle and dietary changes with or without the addition of pharmaceuticals. It has already been shown that these medical treatments are not comparable in effectiveness to bariatric surgery. In type 2 diabetes, the Look Ahead study showed that cardiovascular risk is only reduced with a weight loss of at least 10% of body weight and most medical treatment programs do not lead to mean weight loss of that magnitude [35]. Programs that involve very low-calorie diets, however, can result in large weight loss as well as diabetes remission, as recently shown by the DiRECT study [36]. Such treatment might therefore be more comparable to bariatric surgery in the short-term. The main criticism of LED or VLED has been that the weight loss after longer follow-up than 12 months is not well documented. The medical treatment arm in BASUN is, however, a combination of LED, dietary advice under the food reintroduction period, as well as the possible addition or adjustment of pharmaceutical treatment. In Sweden, the same supplement treatment is recommended for RYGB and SG patients postoperatively. A study recently published by Aarts and colleagues confirmed the need for postoperative nutritional management after SG [37] and suggested that larger doses may be required. Earlier studies have focused on nutritional management after malabsorptive surgery [38, 39].

The BASUN study will assist in identifying which factors are important when it comes to the choice of treatment for obesity, and further aim to define responders and non-responders of surgery and medical treatment. Sub-studies will also be initiated, addressing potential long-term adverse effects of bariatric surgery such as osteoporosis, pre- and postoperative nutritional deficiencies, gastrointestinal function, psychiatric disorders and dental health. These have not all been sufficiently studied previously.

## Conclusion

In summary, BASUN seeks to describe in detail short and long-term effects of bariatric surgery and compare the outcomes of currently available surgical and medical treatments for obesity in clinical practice. The ultimate goal is to reduce complications of treatment and focus public health resources where they benefit the patient the most by defining risk patients before the start of treatment.

## Data Availability

Data and material are not available due to the nature of this prospective study. The datasets used and/or analysed during the current study available from the corresponding author on reasonable request.

## Abbreviations

BMI: Body mass index
MT: Medical treatment
RYGB: Roux-en-Y gastric bypass
SG: Sleeve gastrectomy
T2D: Type 2 diabetes
